# Epo/EpoR signaling in osteoprogenitor cells is essential for bone homeostasis and Epo-induced bone loss

**DOI:** 10.1038/s41413-021-00157-x

**Published:** 2021-09-13

**Authors:** Martina Rauner, Marta Murray, Sylvia Thiele, Deepika Watts, Drorit Neumann, Yankel Gabet, Lorenz C. Hofbauer, Ben Wielockx

**Affiliations:** 1 Department of Medicine III & Center for Healthy Aging, Technische Universität Dresden, Dresden, Germany; 2 Institute for Clinical Chemistry and Laboratory Medicine, Technische Universität Dresden, Dresden, Germany; 3 Department of Cell and Developmental Biology, Sackler Faculty of Medicine, Tel Aviv University, Tel Aviv, Israel; 4 Department of Anatomy & Anthropology, Sackler Faculty of Medicine, Tel Aviv University, Tel Aviv, Israel

**Keywords:** Bone, Pathogenesis, Endocrine system and metabolic diseases

## Abstract

High erythropoietin (Epo) levels are detrimental to bone health in adult organisms. Adult mice receiving high doses of Epo lose bone mass due to suppressed bone formation and increased bone resorption. In humans, high serum Epo levels are linked to fractures in elderly men. Our earlier studies indicated that Epo modulates osteoblast activity; however, direct evidence that Epo acts via its receptor (EpoR) on osteoblasts in vivo is still missing. Here, we created mice lacking EpoR in osteoprogenitor cells to specifically address this gap. Deletion of EpoR in osteoprogenitors (*EpoR:Osx-cre*, cKO) starting at 5 weeks of age did not alter red blood cell parameters but increased vertebral bone volume by 25% in 12-week-old female mice. This was associated with low bone turnover. Histological (osteoblast number, bone formation rate) and serum (P1NP, osteocalcin) bone formation parameters were all reduced, as were the number of osteoclasts and TRAP serum level. Differentiation of osteoblast precursors isolated from cKO versus control mice resulted in lower expression of osteoblast marker genes including Runx2, Alp, and Col1a1 on day 21, whereas the mineralization capacity was similar. Moreover, the RANKL/OPG ratio, which determines the osteoclast-supporting potential of osteoblasts, was substantially decreased by 50%. Similarly, coculturing cKO osteoblasts with control or cKO osteoclast precursors produced significantly fewer osteoclasts than coculture with control osteoblasts. Finally, exposing female mice to Epo pumps (10 U·d^−1^) for 4 weeks resulted in trabecular bone loss (−25%) and increased osteoclast numbers (1.7-fold) in control mice only, not in cKO mice. Our data show that EpoR in osteoprogenitors is essential in regulating osteoblast function and osteoblast-mediated osteoclastogenesis via the RANKL/OPG axis. Thus, osteogenic Epo/EpoR signaling controls bone mass maintenance and contributes to Epo-induced bone loss.

## Introduction

Erythropoietin (Epo) is a kidney-produced hormone that effectively stimulates erythropoiesis. Thus, it is also used clinically to stimulate erythropoiesis in anemia resulting from chronic kidney disease, myelodysplastic syndromes, or cancer.^1–4^ Epo signals via its receptor EpoR.^5,6^ Deletion of Epo or EpoR in mice leads to embryonic lethality due to severe anemia, underscoring the relevance of Epo-EpoR signaling in red blood cell production. Accumulating evidence shows, however, that Epo also exerts extraerythropoietic activities in a variety of tissues. As such, Epo supports the formation of vessel networks, protects against myocardial infarction and ischemic brain injuries, and promotes neural cell proliferation and viability, and it improves energy homeostasis via effects on skeletal muscle cells, hepatocytes, and adipocytes.^7–11^ Another tissue that is heavily influenced by Epo is the skeleton, particularly bone tissue.^12–18^ However, how Epo influences bone mass and whether direct and/or indirect actions on bone cells are involved are matters of ongoing debate. Given the frequent use of Epo in patients who already have compromised bone health, understanding its impact on bone is of central importance.

Endogenous overexpression of Epo or exogenous administration of high doses of Epo reduces bone mass in adult mice.^10,12,15,17,19^ The interpretation of the underlying cellular mechanisms has been controversial; most often, however, increased bone resorption and decreased bone formation have been described. In line with this, high levels of serum Epo have been shown to predict incident fractures in elderly men with normal kidney function.^20^ In young mice, Epo appears to have anabolic effects on bone remodeling in growth and repair by promoting osteoblast differentiation.^14,16,18,21^ Studies have shown that Epo can induce osteoblastogenesis via mTOR and Ephrin B2/EphB4 signaling.^13,14^ In addition, in a study of 60 patients with tibiofibular fractures, local Epo administration promoted bone healing.^22^ Both mature osteoblasts and, to a lesser extent, preosteoclasts express EpoR mRNA transcripts.^12,15^ Recently, Suresh et al. showed that mice with erythroid-restricted EpoR expression exhibited decreased bone mass due to increased numbers of osteoclasts, while osteoblasts were investigated only ex vivo and showed a decreased differentiation capacity.^23^ These mice were also protected from Epo-induced bone loss, suggesting that bone loss due to Epo is mediated by nonerythroid cells.^23^ A follow-up study by this group has now reported on mice lacking EpoR in mature osteoblasts using osteocalcin-cre and has similarly found that female EpoR cKO mice are protected from Epo-induced bone loss.^24^

In this study, we generated mice lacking EpoR in osteoprogenitors using the osterix-cre line, as this construct also targets earlier osteogenic cell populations, including osteoprogenitors. Our data show that female mice with EpoR deficiency in osteoprogenitors exhibit a low bone formation rate and high bone mass due to hampered osteoclastogenesis via the RANKL/OPG pathway. Similarly, this lack of osteoclastogenic support by EpoR-deficient osteoblasts confers osteo-protection from Epo-induced bone loss. Taken together, our study results indicate that EpoR signaling in osteoprogenitors is essential for osteoblast function and osteoblast-to-osteoclast crosstalk via the RANKL/OPG system, thereby regulating adult bone remodeling under physiological and pathophysiological conditions.

## Results

### Conditional loss of EpoR in osteogenic cells increases bone mass and suppresses bone turnover in female but not male mice

**Fig. 1:**
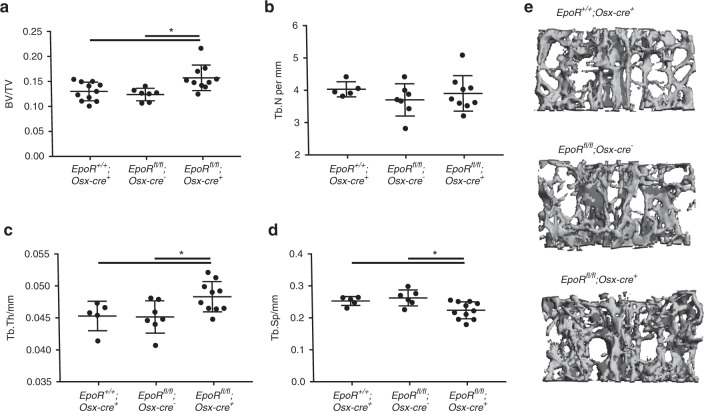
Deletion of EpoR using Osx-cre increases bone mass. Micro-CT measurements were performed at the fourth lumbar vertebra of 10- to 12-week-old female mice. **a** Quantification of the bone volume/total volume (BV/TV) ratio, **b** trabecular number (Tb.N), **c** trabecular thickness (Tb.Th), and **d** trabecular separation (Tb.Sp). Each dot indicates an individual mouse. The horizontal line represents the mean. **e** Representative µCT images of the fourth lumbar vertebra. *N* = 7–11 per group. *P < 0.05

To investigate whether EpoR has direct roles in osteoblast differentiation and/or function and bone mass maintenance, we knocked out EpoR in osteoprogenitor cells from 5 weeks of age using Osx-cre. EpoR deficiency was confirmed at the mRNA level in osteoblast cultures but did not result in a change in body weight compared to WT expression (Supplementary Fig. 1A, B). Moreover, cKO mice exhibited red blood cell parameters, plasma EPO levels, and platelet counts similar to those of their WT littermate controls (Supplementary Fig. 2A–E). Female cKO mice displayed a higher bone volume than both types of control mice, *EpoR*^*fl/fl*^*;Osx-cre*-negative mice and *EpoR*^*wt/wt*^*;Osx-cre*-positive mice (Fig. 1a). There was no difference in bone volume between the two control groups. While trabecular number was not altered (Fig. 1b), cKO mice had thicker trabeculae (Fig. 1c) and decreased trabecular separation (Fig. 1d). Representative images are shown in Fig. 1e. Male cKO mice did not show a difference in bone volume, although some microarchitectural differences in their bone tissue relative to that of controls were observed (Supplementary Fig. 3A–D).

**Fig. 2:**
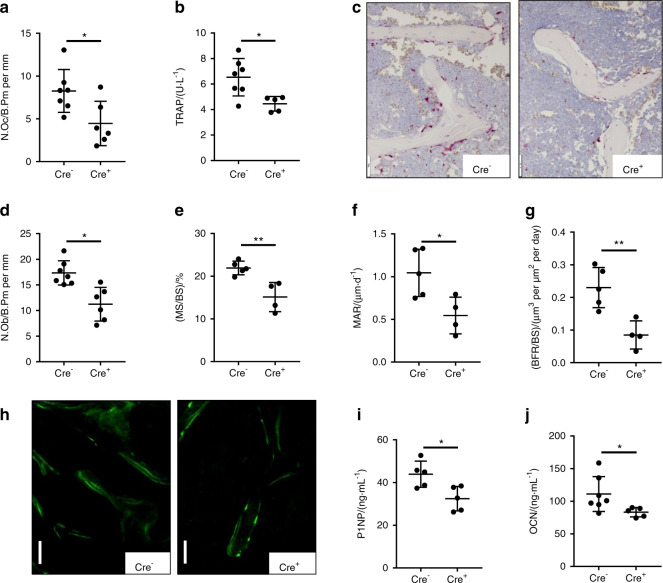
Osteogenic deletion of EpoR results in low bone turnover. Serum markers of bone turnover and parameters for dynamic histomorphometry of the third and fourth lumbar vertebrae of 10- to 12-week-old *EpoR*^*fl/fl*^;*Osx-cre*-negative and *EpoR*^*fl/fl*^;*Osx-cre*-positive mice. **a** Number of osteoclasts per bone perimeter (N.Oc/B.Pm) determined by histology and TRAP staining. **b** Serum tartrate-resistant acid phosphatase (TRAP). **c** Representative TRAP-stained images of Cre^−^ and Cre^+^ mice. Scale bar: 50 µm. **d** Number of osteoblasts per bone perimeter (N.Ob/B.Pm). **e**–**g** Mineralizing surface/bone surface (MS/BS) ratio, mineral apposition rate (MAR), and bone formation rate/bone surface (BFR/BS) ratio. **h** Representative images of calcein labels. Scale bar: 50 µm. **i** Serum osteocalcin (OCN) and **j** N-terminal propeptide of type I procollagen (P1NP). Each dot indicates an individual mouse. The horizontal line represents the mean. *N* = 4–7 per group. *P < 0.05*,* **P < 0.01

To explore whether the increased bone mass in female mice was due to increased bone formation or decreased bone resorption, we performed dynamic bone histomorphometry and assessed serum levels of bone turnover markers. The number of osteoclasts per bone perimeter was twofold lower in cKO mice than in WT mice (Fig. 2a). Similarly, the serum levels of tartrate-resistant acid phosphatase (TRAP), a marker of osteoclast numbers, were also lower (Fig. 2b). Representative images of osteoclasts in histological sections of vertebrae are shown in Fig. 2c. Interestingly, parameters of bone formation were similarly lower in cKO mice than in control mice. The number of osteoblasts per bone perimeter was decreased by 32% (Fig. 2d), and the mineralized surface/bone surface (MS/BS) ratio (Fig. 2E), mineral apposition rate (MAR) (Fig. 2f), and bone formation rate (Fig. 2g) were lower. Representative images of calcein double labeling are shown in Fig. 2h. Both serum markers of bone formation, N-terminal propeptide of type I procollagen (P1NP) and osteocalcin (OCN), were also significantly lower (Fig. 2i, j). Taken together, these results suggest that EpoR deficiency in osteoprogenitors reduces their activity and potentially impairs their crosstalk with osteoclasts, resulting in decreased numbers of osteoclasts, reduced bone resorption, and subsequently higher bone mass.

### Loss of EpoR in osteoblasts leads to reduced expression of osteoblast marker genes but does not change the mineralization capacity in vitro

**Fig. 3:**
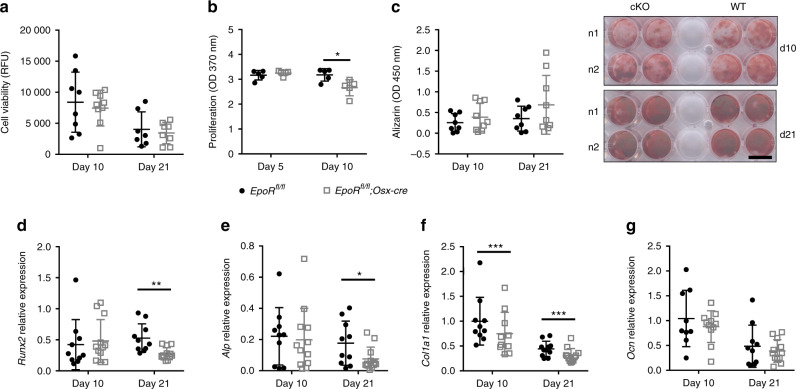
EpoR-deficient osteoblasts show lower expression of osteoblastic marker genes but normal mineralization properties. Osteoblasts were derived by differentiating bone marrow stromal cells from 10- to 12-week-old WT and cKO mice. After 10 and 21 days, various assays were performed. **a** Cell viability measured via CellTiter Blue. **b** Cell proliferation measured using BrdU incorporation on day 5 and 10 of differentiation. *N* = 5 per group. **c** Alizarin red staining and representative images from each condition/genotype. N1–2 indicates individual mice. Scale bar: 15 mm. **d**–**g** mRNA levels of osteoblast markers determined via qPCR. Data are normalized to the level of β-actin. Each dot indicates an individual mouse. The horizontal line represents the mean. *N* = 8–9 per group. *P < 0.05*,* **P < 0.01*,* ***P < 0.001

To gain insights into the role of EpoR during osteoblast differentiation, we differentiated osteoblasts from the bone marrow of cKO and WT littermates. After 10 and 21 days of differentiation, cKO and WT osteoblasts were viable (Fig. 3a). Cell proliferation was not altered 5 days after differentiation but was decreased significantly (by 16%) in osteoblasts from cKO mice after 10 days of differentiation (Fig. 3b). Moreover, the amount of mineralized matrix did not differ between cKO and WT mice (Fig. 3c). Interestingly, osteoblast marker gene expression was reduced in cKO mice, especially during the later stages of differentiation (Fig. 3d–g). *Runx2* and *ALP* mRNA levels were reduced by 48% and 57%, respectively, in cKO osteoblasts compared to WT osteoblasts on day 21, while no change was detectable on day 10 (Fig. 3d, e). The expression levels of type 1 collagen were significantly lower at both time points (Fig. 3f). *OCN* mRNA levels, on the other hand, were not affected by EpoR deficiency (Fig. 3g). Thus, even though the gene expression levels of the main osteoblast markers are reduced in osteoblasts lacking EpoR, this does not appear to have a major effect on the mineralization capacity of these osteoblasts in vitro.

### EpoR in osteoblasts controls OPG expression and osteoclast differentiation

**Fig. 4:**
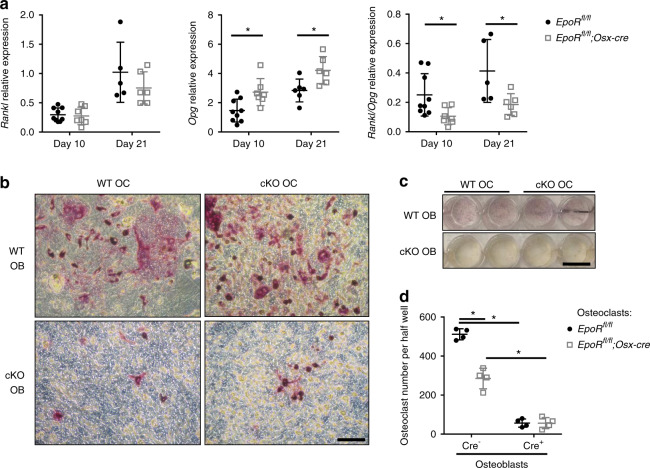
EpoR-deficient osteoblasts display a lower RANKL/OPG ratio and thus fail to support osteoclastogenesis. **a** Osteoblasts were differentiated from the bone marrow of WT and cKO mice for 10 or 21 days. Afterwards, the mRNA expression of RANKL and OPG was assessed (normalized to that of β-actin). The RANKL/OPG ratio is shown. **b**–**d** Osteoblast-osteoclast coculture was performed using WT or cKO calvarial osteoblasts and/or bone marrow-derived osteoclasts. After 8 days of coculture, images were acquired under a microscope (scale bar: 0.1 mm) (**b**) and from the wells (scale bar: 15 mm) (**c**). **d** Quantification of osteoclast numbers per well. Each dot indicates an individual mouse/coculture condition. The horizontal line represents the mean. *N* = 8–9 per group. *P < 0.05

To investigate how EpoR in osteoblasts might regulate osteoclast differentiation, we analyzed the mRNA expression of RANKL and OPG, two important cytokines that control osteoclastogenesis. While EpoR deficiency did not result in significantly reduced RANKL mRNA levels, OPG expression was highly increased in osteoblasts from cKO mice at both 10 and 21 days (Fig. 4a). This resulted in a markedly lower RANKL/OPG ratio at both 10 and 21 days (Fig. 4a). Of note, RANKL expression was not significantly altered in bone tissue isolated from cKO mice (WT: 21.7 ± 9.2 vs. cKO 30.2 ± 12.5 relative expression, *P* = 0.23), while OPG expression was upregulated (WT: 12.6 ± 5.8 vs. cKO 36.8 ± 21.6 relative expression, *P* < 0.05), resulting in a decreased RANKL/OPG ratio in bone tissue (WT: 1.75 ± 0.27 vs. cKO 0.95 ± 0.51, *P* < 0.01).

To investigate whether this altered RANKL/OPG ratio affects osteoclast differentiation, we performed osteoblast-osteoclast cocultures. Interestingly and in line with our in vivo data, cKO osteoblasts gave rise to notably fewer osteoclasts than WT osteoblasts, regardless of the genotype of the osteoclast precursors (Fig. 4b–d). Moreover, fewer osteoclasts differentiated in cocultures with WT osteoblasts if the osteoclast precursors were derived from cKO mice rather than WT mice (Fig. 4b–d).

To exclude possible direct effects on osteoclasts, we differentiated osteoclasts from the bone marrow of cKO and WT mice. On day 6 of differentiation, EpoR mRNA levels were not different between cKO and WT mice (Supplementary Fig. 4A). In addition, osteoclast numbers and the expression of osteoclast-specific genes including TRAP, osteoclast-associated receptor, and cathepsin K were not different (Supplementary Fig. 4B, C), suggesting no off-target effects of osteogenic EpoR deletion on osteoclasts.

### Epo-induced trabecular bone loss in adult female mice is at least partly mediated via EpoR in osteoblasts

**Fig. 5:**
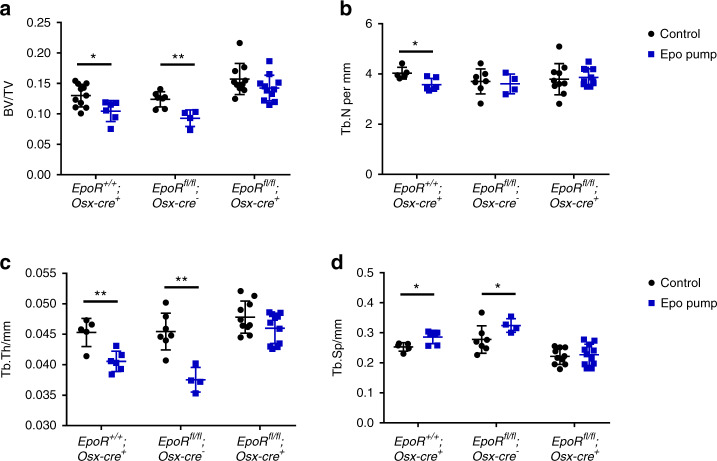
Mice deficient in EpoR in osteoblasts are partially protected from Epo-induced bone loss. Micro-CT analysis of the fourth lumbar vertebra of 10-week-old female cKO and WT littermates that were treated with Epo pumps (10 U) for 4 weeks. **a** Bone volume/total volume (BV/TV) ratio, **b** trabecular number (Tb.N), **c** trabecular thickness (Tb.Th), and **d** trabecular separation (Tb.Sp). Each dot indicates an individual mouse. The horizontal line represents the mean. *N* = 4–11 per group. *P < 0.05, **P < 0.01

**Fig. 6:**
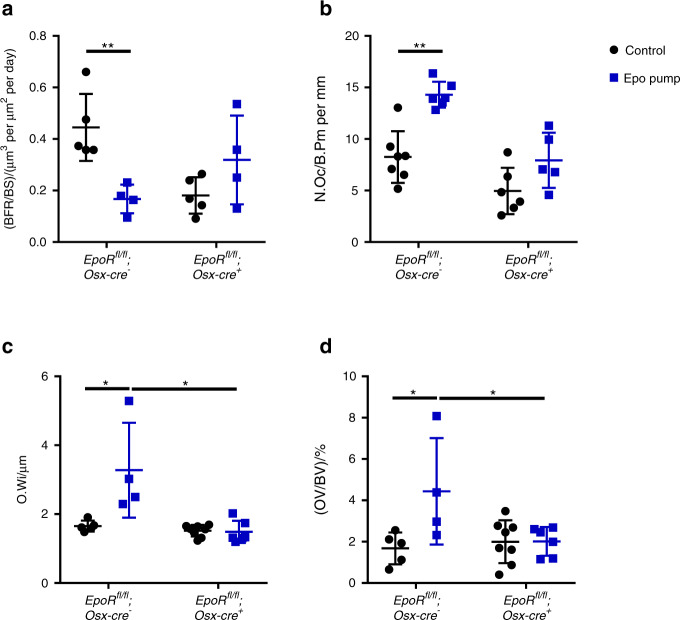
Bone turnover in Epo-treated mice deficient in EpoR in osteoblasts. Dynamic bone histomorphometry was performed on the fourth lumbar vertebra of 10-week-old female cKO and WT mice treated with Epo (10 U) for 4 weeks. **a** Bone formation rate/bone surface (BFR/BS), **b** osteoclast number/bone perimeter (N.Oc/B.Pm), **c** osteoid width (O.Wi), and **d** osteoid volume/bone volume (OV/BV). Each dot indicates an individual mouse. The horizontal line represents the mean. *N* = 4–6 per group. **P < 0.01.

As our previous work strongly suggested the impact of osteoblast-dependent Epo/EpoR signaling in mice chronically overexpressing Epo,^15^ we wanted to directly address this assumption in vivo. Thus, we subjected cKO mice to a 4-week chronic treatment with Epo via osmotic pumps. As previously reported, WT mice subjected to this protocol lost bone volume and showed a deteriorated bone microarchitecture (Fig. 5a–d).^15^ Bone loss occurred due to reduced numbers of osteoblasts (Fig. 6a) and increased numbers of osteoclasts (Fig. 6b). In addition, the formation of osteoids was increased, as indicated by thickened osteoid seams and an increased osteoid volume (OV) (Fig. 6c, d). The same was true for *EpoR*^*wt/wt*^;*Osx-cre*-positive mice (Fig. 5a–d). In contrast, cKO mice were protected from Epo-induced bone loss and deterioration of the bone microarchitecture (Figs. 5a–d and 6c, d). In line with our previous study,^15^ these changes appear to be the result of a lack of suppression of the bone formation rate and the formation of osteoids as well as reduced induction of osteoclast numbers by Epo in cKO mice (Fig. 6a–d).

## Discussion

We and others have previously unambiguously shown that the administration of high doses of Epo to adult mice or transgenic overexpression of Epo induces bone loss.^10,12,15,17,19,23,24^ As osteoclastic bone resorption is increased and osteoblastic bone formation is reduced by Epo administration, both cell types could contribute to Epo-induced bone loss. As our previous study^15^ and more recent studies^23,24^ strongly suggest that Epo/EpoR signaling in osteogenic cells contributes to Epo-induced bone loss, we aimed to test this formally in vivo using mice lacking EpoR in osteoprogenitor cells. This mouse model further allowed us to investigate the role of EpoR signaling in osteolineage cells during bone homeostasis. Our results show that EpoR in osteoprogenitors is crucial for proper osteoblast function in vivo, as demonstrated by the reduced bone formation rate and low concentrations of serum bone formation markers, and that perhaps more importantly, EpoR in osteoprogenitors stimulates osteoclastogenesis via the RANKL/OPG system. Loss of EpoR in osteoblasts results in a dramatic inhibition of osteoclastogenesis, leading to a high bone mass phenotype and to protection from Epo-induced bone loss in adult mice.

Previously, Suresh et al. showed that EpoR in nonerythroid cells, more specifically in mature osteoblasts (using the osteocalcin-cre line), contributes to Epo-induced bone loss and bone remodeling in adult mice.^23,24^ However, in contrast to our model, these nonerythroid EpoR-deficient mice showed a low bone mass phenotype. Interestingly, these mice showed more severe bone loss in males and females under steady-state conditions than mice lacking EpoR in mature osteoblasts only. In fact, trabecular bone loss of approximately 10%–15% was observed in only male *EpoR:Ocn-cre* mice, not females. Interestingly, by histological analysis, mice lacking EpoR in mature osteoblasts showed equal numbers of osteoblasts and osteoclasts compared to their littermate controls.^24^ These data suggest that EpoR on cells other than mature osteoblasts may contribute to bone mass maintenance. Our study now provides evidence that earlier cells of the osteolineage that are targeted by the Osx-cre construct may represent such cells. It should be noted, however, that Osx-cre also targets cells other than osteoprogenitors, such as hypertrophic chondrocytes, perivascular cells, and bone marrow adipocytes, suggesting that these cells may also contribute to the phenotype.^25^ Finally, it is interesting to note that EPO treatment caused an increase in osteoids in wild-type animals, which may be due to the induction of the phosphaturic hormone fibroblast growth factor-23 by Epo.^26,27^ The lack of an increase in osteoids during Epo treatment in *EpoR:Osx-cre* mice suggests that the decreased mineralization ability of these osteoblasts potentially protects them from overactivation and production of unmineralized bone.

In addition to decreased osteoblast numbers and function in vivo, mice lacking EpoR in osteoprogenitors showed decreased numbers of osteoclasts and lower serum levels of the osteoclast marker TRAP, strongly suggesting that osteoblast-to-osteoclast crosstalk was impaired. Using a coculture approach, we revealed that osteoblasts lacking EpoR expressed high mRNA levels of OPG, an osteoclast inhibitory protein, thus leading to impaired support of osteoclastogenesis. This is the first study to show that EpoR affects OPG expression and that the lack of osteoclastogenesis induction in chronically Epo-treated *EpoR:Osx-cre* mice appears to be the main mechanism conferring osteo-protection in these mice. These results are in line with the findings that mice deficient in EpoR in mature osteoblasts were protected from Epo-induced bone loss,^24^ although the underlying cellular mechanisms of osteo-protection in *EpoR:Ocn-cre* mice^24^ have not been fully resolved. Finally, another cell type that may drive Epo-induced bone loss is B cells. Even though B cells are reduced in mice after Epo treatment,^17^ we showed that Epo induces RANKL expression in B cells to stimulate osteoclastogenesis.^28^ As such, mice with EpoR-deficient B cells are also partially protected from Epo-induced bone loss.^28^ Thus, these studies suggest that various cell types in the bone marrow niche collectively contribute to the enhanced osteoclastogenesis observed during Epo treatment. Determining whether Epo also stimulates osteoclasts directly via EpoR in vivo will provide important information for understanding the cellular mechanisms of Epo-induced bone loss.

In the current study and in a study by Suresh et al.,^24^ females showed more pronounced protection from Epo-induced bone loss than males. Of note, EpoR also shows sexual dimorphic features in other tissues, including the brain and adipose tissue.^29,30^ Studies in breast cancer suggest a close correlation between estrogen receptor and EpoR,^31^ indicating that these receptors may interact with each other. More studies are clearly needed to dissect this relationship and address the sexual dimorphic effects of the Epo-EpoR axis on bone.

In summary, our data indicate that Epo/EpoR signaling is required for proper osteogenic differentiation and osteoblast-to-osteoclast crosstalk, thereby playing important roles in physiological bone remodeling and Epo-induced bone loss.

## Methods

### Experimental animals

The *Osx:cre* transgenic mouse line^32^ was obtained from The Jackson Laboratory (Bar Harbor, ME, USA) and crossed with *EpoR*^*f/f*^ mice^33^ in our facility. These mice are referred to as cKO mice. Breeding pairs continuously received doxycycline (dox) in the drinking water (10 mg·mL^−1^ dox in a 3% w/v sucrose solution) ad libitum, including during pregnancy. All offspring were kept with their parents until they reached the age of 5 weeks. After weaning, all offspring received normal drinking water. Cre-negative *EpoR*^*f/f*^ littermate mice were used as controls (WT). In addition, due to previous results showing skeletal defects in growing *Osx:cre*-positive mice,^34^ *EpoR*^*wt/wt*^;Osx:cre-positive mice receiving the same dox treatment were analyzed. However, their bone density was not significantly different from that of *EpoR*^*f/f*^;*Osx:cre*-negative mice.

Osmotic pumps (Alzet^®^, Cupertino, CA, USA) filled with recombinant human Epo (10 U·d^−1^) were transplanted on the back of mice underneath the skin and maintained for 30 days.^15^ All experiments were performed with male and female mice between the ages of 10 and 12 weeks in a randomized fashion or as indicated in the text. Mice were maintained in groups of up to four animals per cage and kept on a 12-h light/dark cycle. Water and food were available ad libitum. Mice were allocated into groups on availability of the conditional deficient transgenic mice and/or their control littermates.

### Ethical statement

Mice were anesthetized with a single injection of ketamine (100 mg·kg^−1^)/xylazine (10 mg·kg^−1^). Before the final analysis, mice were euthanized by cervical dislocation. All experiments were conducted at the Medical Theoretical Centre of the Medical Faculty, TU Dresden, Germany. All animal experiments were in accordance with the facility guidelines on animal welfare and approved by the Landesdirektion Sachsen, Germany (approval numbers: TVV 09/2014, 15/2017, and 12/2019).

### Blood analysis

Blood parameters (hematocrit, RBCs, Hb, and thrombocytes) were measured using a Sysmex automated blood cell counter (Sysmex XE-2100 and XE-5000). The plasma EPO level was determined using the Quantikine Mouse/Rat EPO immunoassay (R&D Systems).

### Bone structure analysis and histomorphometry

µCT and bone histology were performed as described previously.^35^ Briefly, the entire fourth lumbar vertebra was scanned at an isotropic voxel size of 10.5 µm with an integration time of 200 ms, X-ray intensity of 140 µA, and energy of 70 kV (vivaCT40, Scanco Medical AG, Switzerland). Within vertebrae, 100 slices (50 above and 50 below the center) were contoured to evaluate the trabecular bone structure using predefined scripts from Scanco.

For bone histomorphometry, mice received two intraperitoneal injections of calcein (20 mg·kg^−1^, Sigma, Germany) on day 5 and 2 before sacrifice. After collection, the third lumbar vertebra was dissected, fixed in 4% PBS-buffered paraformaldehyde for 24 h and dehydrated in an ascending ethanol series. Subsequently, bones were embedded in methacrylate and cut into 7-µm sections. Unstained sections were analyzed using fluorescence microscopy to determine the MS/BS ratio, the MAR, and the bone formation rate/bone surface ratio. To assess OV and osteoid width (O. Wi), 4-μm plastic sections were stained with von Kossa/van Gieson stains.

Osteoblast and osteoclast parameters were further determined with TRAP-stained paraffin sections from the fourth vertebral body. To that end, bones were first demineralized for 1 week in Osteosoft (Merck, Germany). Histomorphometric analysis was performed with Osteomeasure software (OsteoMetrics, Decatur, GA, USA) according to international standards.

### Osteoblast culture and cell viability

Harvesting of bone marrow stromal cells from the long bones of 10- to 12-week-old mice was performed by cutting the bones (femur and tibia) at both ends and flushing out the bone marrow using DMEM. Cells were centrifuged, resuspended in DMEM + 10% FCS + 1% P/S and plated in multiwell dishes at a density of 10^6^ cells per cm^2^. After 5 days, the medium was changed. After reaching 70% confluence, the cells were switched to osteogenic medium containing 100 μmol·L^−1^ ascorbate phosphate and 5 mmol·L^−1^ β-glycerophosphate. After 5 and 10 days, cell proliferation was assessed using a BrdU assay (Roche) according to the manufacturer’s instructions. After 10 and 21 days, RNA was isolated for gene expression analysis, and the mineralized matrix was stained with alizarin red S (1%, Sigma-Aldrich). Alizarin red S was eluted with 100 mmol·L^−1^ cetylpyridinium chloride and quantified using a spectrophotometer at 540 nm. Cell viability was measured on day 10 and 21 using CellTiter Blue (Promega).

### Osteoblast-osteoclast coculture

Osteoblasts were generated from 3- to 5-day-old cKO and WT mice from breeding pairs that received drinking water containing dox. Immediately after calvarial isolation, mice were genotyped to separately pool 4–6 cKO and 4–6 WT mice. Calvaria were pooled and digested five times for 10 min at 37 °C with a 0.2% collagenase and 0.15% dispase solution. Cells were plated at a density of 2 × 10^4^ cells per mL alpha-MEM supplemented with 10% FCS and 1% P/S in a 24-well plate. After 3 days, the cells were switched to differentiation medium containing 50 μmol·L^−1^ ascorbate phosphate and 5 mmol·L^−1^ β-glycerol phosphate. In parallel, bone marrow cells were obtained from adult cKO and WT mice by flushing the bone marrow. r of 10^6^ cells per cm^2^ in alpha-MEM supplemented with 10% FCS, 1% P/S, and 100 ng·mL^−1^ M-CSF. After 3 days of bone marrow cell culture, which was day 6 of calvarial osteoblast culture, 2 × 10^5^ bone marrow macrophages were added to osteoblasts in alpha-MEM containing 10% FCS, 1% P/S, 10 nmol·L^−1^ 1,25-vitamin D_3_, and 1 μmol·L^−1^ PGE_2_. After another 3 days, the medium was changed, and after 8 additional days, the cells were fixed and stained for TRAP using the Acid Phosphatase, Leukocyte (TRAP) Kit from Sigma.

### Osteoclast culture

Using the same bone marrow cells as described for the coculture assay, osteoclasts were differentiated from bone marrow cells (10^6^ cells per cm^2^) using 25 ng·mL^−1^ M-CSF (R&D Systems). After 2 days, 50 ng·mL^−1^ RANKL (R&D Systems) was added. After a total of 6 days, cells were stained with TRAP to identify and quantify osteoclasts. In addition, RNA was isolated to analyze the expression of osteoclast-specific genes.

### RNA isolation, RT, and qPCR

For gene expression analysis, RNA was isolated from day 7 and day 21 osteoblasts or osteoclast progenitors using the High Pure RNA Isolation Kit (Roche, Mannheim, Germany) and reverse transcribed using Superscript II (Invitrogen). For analysis of gene expression in bone tissue, bone marrow was flushed from the femur and tibia, and the cortical bone parts were crushed in liquid nitrogen using a mortar and pestle. The bone powder was taken up in TriFast reagent (Peqlab), and RNA was extracted according to the manufacturer´s protocol. mRNA expression was determined by Applied Biosystems SYBR green-based real-time PCR using a standard protocol (ABI 7500Fast, Applied Biosystems, Darmstadt, Germany). The primer sequences were: β-actin sense (s): ATCTGGCACCACACCTTCT, β-actin antisense (as): GGGGTGTTGAAGGTCTCAAA; EpoR s: AGACTTGGTGTGTTTCTGGGAG, EpoR as: TGGTGCAGGCTACATGACTTTC; Runx2 s: CCCAGCCACCTTTACCTACA, Runx2 as: TATGGAGTGCTGCTGCTGGTCTG; ALP s: CTACTTGTGTGGCGTGAAGG, ALP as: CTGGTGGCATCTCGTTATCC; OCN s: GCGCTCTGTCTCTCTGACCT, OCN as: ACCTTATTGCCCTCCTGCTT; Col1a1 s: ACTGTCCCAACCCCCAAAG, Col1a1 as: CGTATTCTTCCGGGCAGAAA; RANKL s: CCAAGATCTCTAACATGACG, RANKL as: CACCATCAGCTGAAGATAGT; OPG s: CCTTGCCCTGACCACTCTTA, OPG as: ACACTGGGCTGCAATACACA; CTSK s: AAGTGGTTCAGAAGATGACGGGAC, CTSK as: TCTTCAGAGTCAATGCCTCCGTTC; TRAP s: ACTTGCGACCATTGTTAGCC, TRAP as: AGAGGGATCCATGAAGTTGC; and OSCAR s: TGGCGGTTTGCACTCTTCA, OSCAR as: GATCCGTTACCAGCAGTTCCAGA. Results were calculated using the ΔΔCT method and are presented relative to the control.

### Serum measurements

Serum levels of the bone formation markers P1NP and OCN and the bone resorption marker TRAP5b were measured using ELISA according to the manufacturer’s protocol (IDS, Frankfurt/Main).

### Statistical analysis

Results are presented as individual dots, each representing one mouse, and the mean is presented as a horizontal line. For comparison of two groups, Student’s *t*-test was performed. For experiments with two factors, two-way ANOVA with the Bonferroni post hoc test was performed. *P* values <0.05 were considered statistically significant (GraphPad Prism).

## Supplementary information

BONERES-01697

## Data Availability

Data and materials will be made available upon request and, if applicable, material transfer agreements.
