# Supplementary figures and images for: Epo/EpoR signaling in osteoprogenitor cells is essential for bone homeostasis and Epo-induced bone loss
Source: Bone Res. 2021 Sep 13;9:42. doi: 10.1038/s41413-021-00157-x (PMC8437981; doi:10.1038/s41413-021-00157-x)

# A

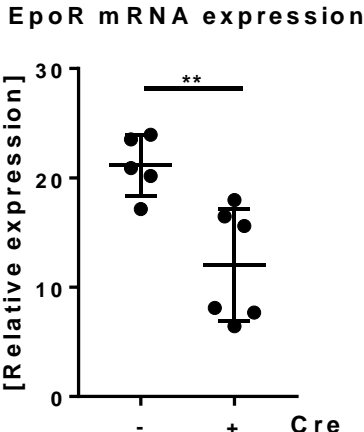

# B

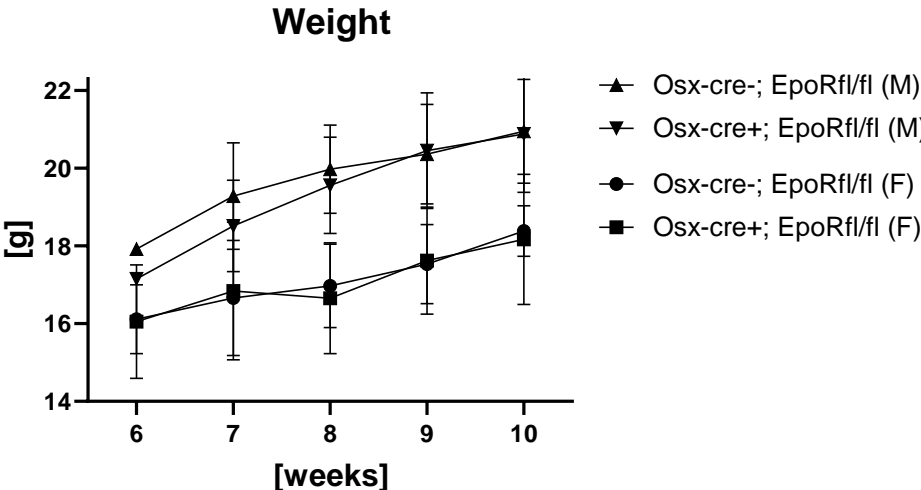

Suppl. Fig. 2

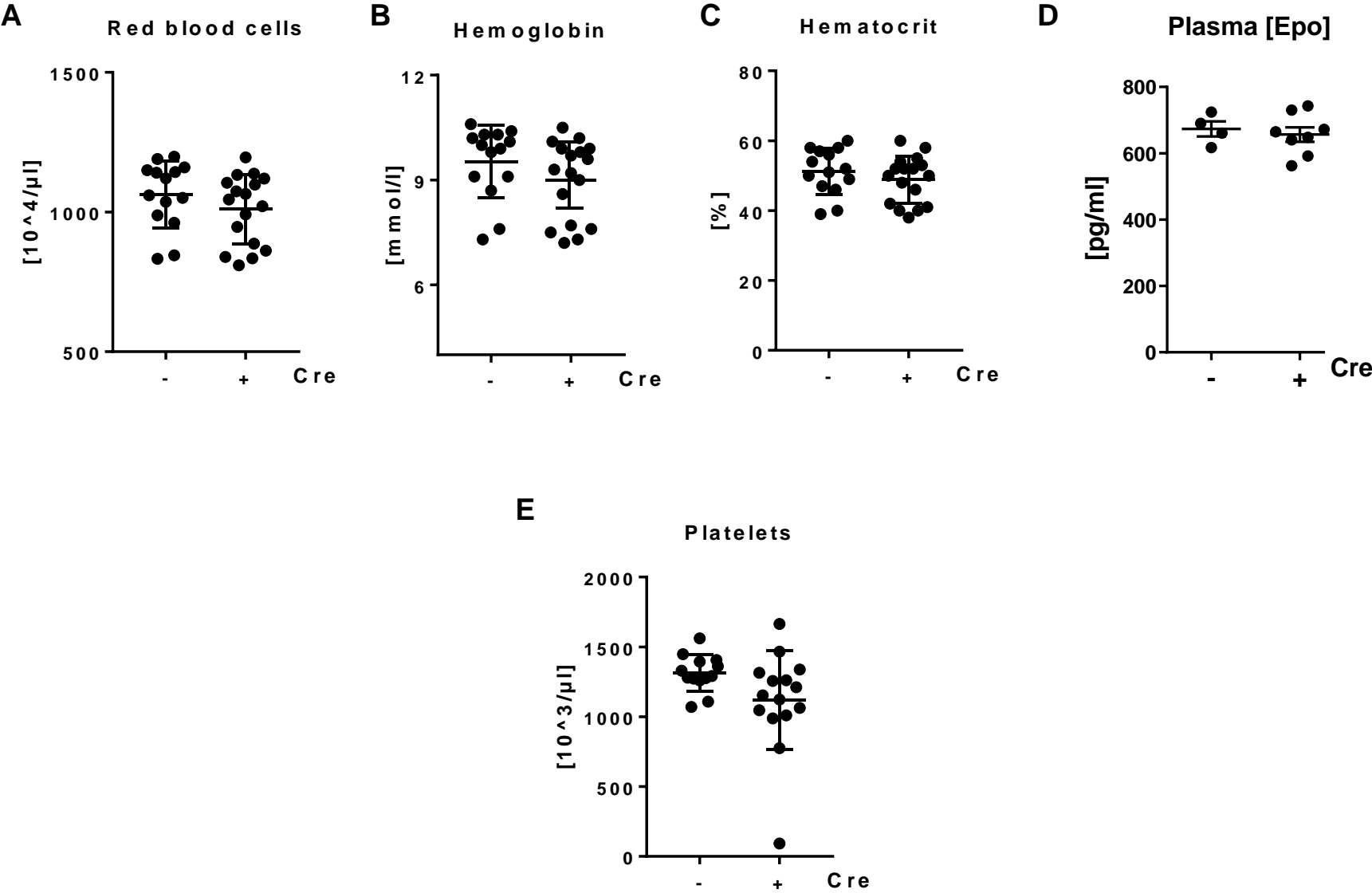

Suppl. Fig. 3

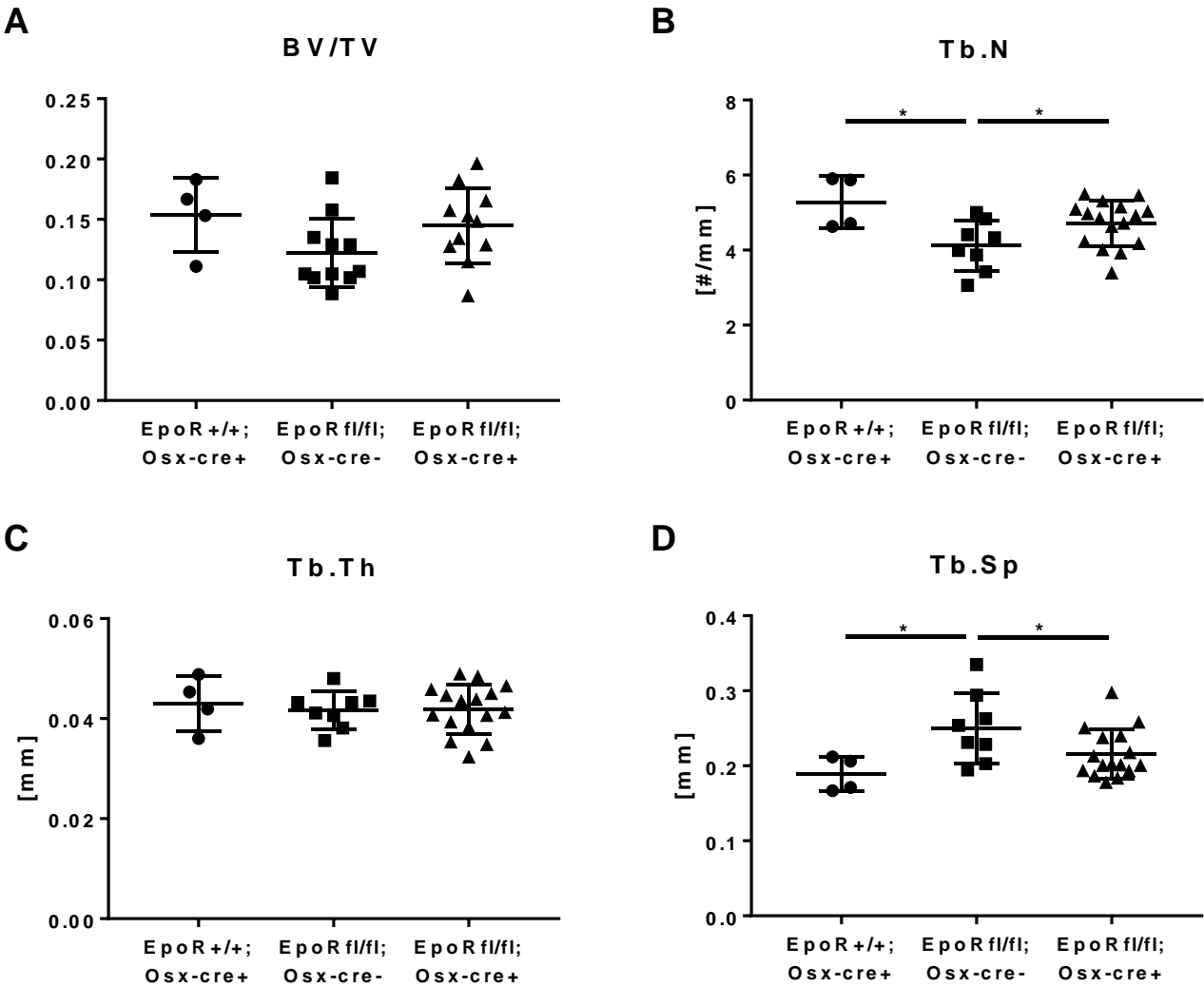

Suppl. Fig. 4

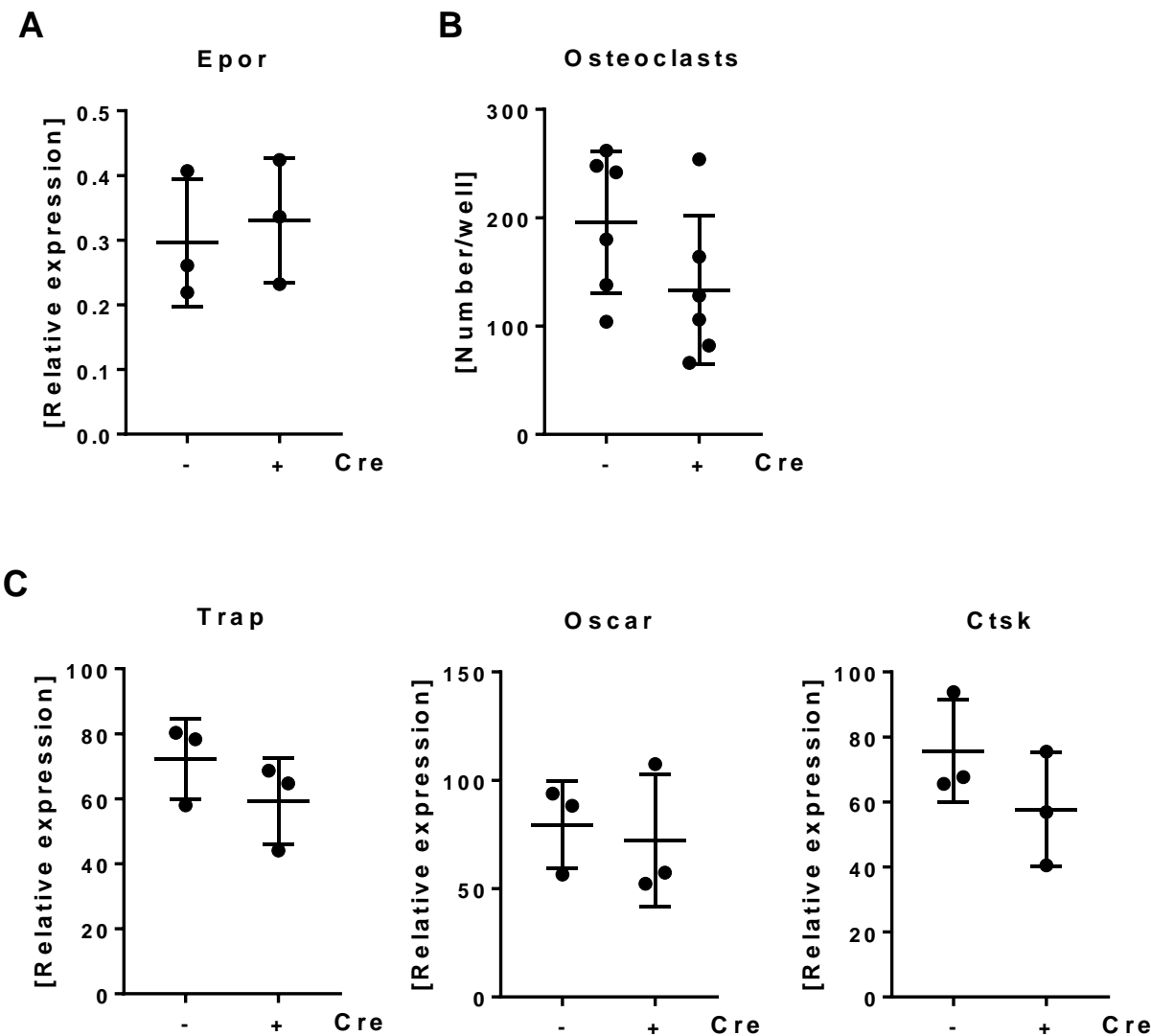

Supplement: Supplementary file 1 — BONERES-01697 [file 41413_2021_157_MOESM1_ESM.pdf]
